# Supplementary material for: Untargeted metabolomics analysis reveals the metabolic disturbances and exacerbation of oxidative stress in recurrent spontaneous abortion
Source: PLoS One. 2023 Dec 21;18(12):e0296122. doi: 10.1371/journal.pone.0296122 (PMC10735046; doi:10.1371/journal.pone.0296122)
Supplement: S1 Table — (DOCX) [file pone.0296122.s001.docx]

**Table S1. Detailed LC-MS information**

| **LC conditions** | | | **MS conditions** | | |
| --- | --- | --- | --- | --- | --- |
| Time(min) | A(%) | B(%) | Term | ESI+ | ESI- |
| 0.0 | 95 | 5 | Duration(min) | 14 | 14 |
| 11.0 | 10 | 90 | IonSpray Voltage(V) | 5000 | -4500 |
| 12.0 | 10 | 90 | Temperature(℃) | 550 | 450 |
| 12.1 | 95 | 5 | Ion Source Gas 1（psi） | 50 | 50 |
| 14.0 | 95 | 5 | Ion Source Gas 2 (psi) | 60 | 60 |
|  |  |  | Curtain Gas (psi) | 35 | 35 |
|  |  |  | Declustering Potential (V) | 60 | -60 |
|  |  |  | MS1 Collision Energy(V) | 10 | -10 |
|  |  |  | MS2 Collision Energy(V) | 30 | -30 |
|  |  |  | Collision Energy Spread (V) | 15 | 15 |
|  |  |  | MS1 TOF Masses (Da) | 50~1000 | 50~1000 |
|  |  |  | MS2 TOF Masses（Da） | 25~1000 | 25~1000 |
|  |  |  | MS1 Accumulation time(s) | 0.2 | 0.2 |
|  |  |  | MS2 Accumulation time(s) | 0.05 | 0.05 |
|  |  |  | Candidate ions | 12 | 12 |
