# Supplementary figures and images for: Untargeted metabolomics analysis reveals the metabolic disturbances and exacerbation of oxidative stress in recurrent spontaneous abortion
Source: PLoS One. 2023 Dec 21;18(12):e0296122. doi: 10.1371/journal.pone.0296122 (PMC10735046; doi:10.1371/journal.pone.0296122)

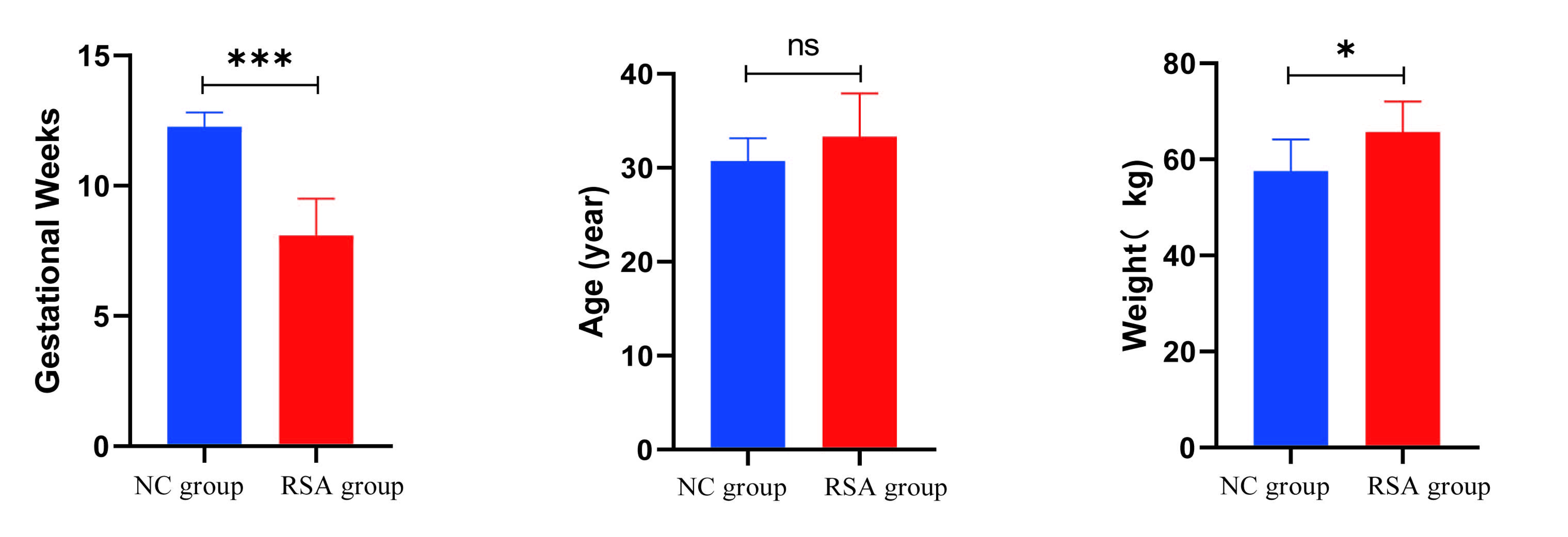

Supplement: S1 Fig — ns: no significance; *: p <0.05; **: p <0.01; ***: p <0.001. (TIF) [file pone.0296122.s005.tif]
