# Supplementary material for: Untargeted metabolomics analysis reveals the metabolic disturbances and exacerbation of oxidative stress in recurrent spontaneous abortion
Source: PLoS One. 2023 Dec 21;18(12):e0296122. doi: 10.1371/journal.pone.0296122 (PMC10735046; doi:10.1371/journal.pone.0296122)
Supplement: Attachment — Submitted filename: Review.docx [file pone.0296122.s006.docx]

It is important to determine gene expression and discuss the RSA at the molecular level.

I suggest joining these findings with the abnormal expression of some specific immunoregulatory genes involved in T-cell activation and differentiation. According to psychophysiological interaction (PPI) analysis, *TLR2, CXCL8, IFNG, IL2RA*, and *ITGAX* were among the top five immunoregulatory hub genes of differentially expressed genes in unexplained recurrent spontaneous abortion (URSA). Among the identified differentially expressed genes (DEGs) in URSA, *IFNG* may play a key role in regulating maternal immune response. AS the key genes and functional pathways identified will provide new insights into the molecular mechanisms involved in RSA pathogenesis and provide potential diagnostic and therapeutic targets.
