# Supplementary material for: Untargeted metabolomics analysis reveals the metabolic disturbances and exacerbation of oxidative stress in recurrent spontaneous abortion
Source: PLoS One. 2023 Dec 21;18(12):e0296122. doi: 10.1371/journal.pone.0296122 (PMC10735046; doi:10.1371/journal.pone.0296122)
Supplement: Attachment — Submitted filename: Review 2.docx [file pone.0296122.s008.docx]

I accepted the article after modifications.
